# An antibiotic stewardship program in a surgical ICU of a resource-limited country: financial impact with improved clinical outcomes

**DOI:** 10.1186/s40545-020-00272-w

**Published:** 2020-10-06

**Authors:** Kashif Hussain, Muhammad Faisal Khan, Gul Ambreen, Syed Shamim Raza, Seema Irfan, Kiren Habib, Hasnain Zafar

**Affiliations:** 1 Department of Pharmacy, Aga Khan University Hospital, Stadium Road (Main Pharmacy), P.O Box 3500, Karachi, 74800 Pakistan; 2 Department of Anesthesia, Aga Khan University Hospital, Karachi, Pakistan; 3 Section of Microbiology, Aga Khan University Hospital, Karachi, Pakistan; 4 Department of Internal Medicine (Infectious Disease), Aga Khan University Hospital, Karachi, Pakistan; 5 Department of Surgery, Aga Khan University Hospital, Karachi, Pakistan

**Keywords:** Antibiotic stewardship program, Antibiotic resistance, Infectious disease pharmacist, Surgical-ICU

## Abstract

**Background:**

Antibiotic resistance (ABX-R) is alarming in lower/middle-income countries (LMICs). Nonadherence to antibiotic guidelines and inappropriate prescribing are significant contributing factors to ABX-R. This study determined the clinical and economic impacts of antibiotic stewardship program (ASP) in surgical intensive care units (SICU) of LMIC.

**Method:**

We conducted this pre and post-test analysis in adult SICU of Aga Khan University Hospital, Pakistan, and compared pre-ASP (September–December 2017) and post-ASP data (April–July 2018). January–March 2018 as an implementation/training phase, for designing standard operating procedures and training the team. We enrolled all the patients admitted to adult SICU and prescribed any antibiotic. ASP-team daily reviewed antibiotics prescription for its appropriateness. Through prospective-audit and feedback-mechanism changes were made and recorded. Outcome measures included antibiotic defined daily dose (DDDs)/1000 patient-days, prescription appropriateness, antibiotic duration, readmission, mortality, and cost-effectiveness.

**Result:**

123 and 125 patients were enrolled in pre-ASP and post-ASP periods. DDDs/1000 patient-days of all the antibiotics reduced in the post-ASP period, ceftriaxone, cefazolin, metronidazole, piperacillin/tazobactam, and vancomycin showed statistically significant (*p* < 0.01) reduction. The duration of all antibiotics use reduced significantly (*p* < 0.01). Length of SICU stays, mortality, and readmission reduced in the post-ASP period. ID-pharmacist interventions and source-control-documentation were observed in 62% and 50% cases respectively. Guidelines adherence improved significantly (*p* < 0.01). Net cost saving is 6360US$ yearly, mainly through reduced antibiotics consumption, around US$ 18,000 (PKR 2.8 million) yearly.

**Conclusion:**

ASP implementation with supplemental efforts can improve the appropriateness of antibiotic prescriptions and the optimum duration of use. The approach is cost-effective mainly due to the reduced cost of antibiotics with rational use. Better source-control-documentation may further minimize the ABX-R in SICU.

## Introduction

Antibiotic resistance (ABX-R) is a big challenge faced worldwide. Moreover, data from lower and middle-income countries (LMICs), is more alarming [1]. With existing challenging issues of over and misuse of antibiotics, deprived sanitation, and infection control practices along with inadequate vaccination are all contributing to increasing the rate of multi-drug resistant infections in LMICs [2]. The inappropriate use of antibiotics is a critically significant however modifiable contributing factor for ABX-R [3]. Studies from developed countries have reported up to 50% of antimicrobial use as inappropriate [4, 5]. Therefore, for improved antibiotics use in hospitalized patients, the antibiotic stewardship program (ASP) has been developed in 2007 and further updated in 2016 by Society for Healthcare Epidemiology of America (SHEA) and Infectious Diseases Society of America (IDSA) [6]. The cornerstone of the ASP is appropriate antibiotics selection and optimization of their doses and duration to ensure the best clinical outcomes, minimal effect on subsequent ABX-R, and toxicity to the patients [7, 8]. The reports of developed countries and the pediatric intensive care unit (PICU) of our hospital had proposed that implementation of ASP can be an effective approach in combating the emergence of ABX-R [9, 10].

There are several advantages of ASP including the reduction in hospital length of stay, optimized treatment duration without increased mortality rate, and lower antimicrobial colonization and resistance [11]. In contrast, less is evaluated about the cost-effectiveness of hospital ASP [11, 12]. Evaluation of this aspect is highly important in the perspective of the financial benefit of ASP in developing countries through minimizing ABX-R. Several studies have been published on ASP implementation in intensive care units (ICUs) and reported significant improvement in antibiotics consumption trends [13–15]. Additionally, the judicious start of appropriate antimicrobial agents at a suitable time has been established to decrease disease severity and deaths, as in the case of sepsis, the timely start of antibiotic therapy is as important as the selection of antibiotics [16]. Usually, there are several reasons for using antibiotics in ICUs due to the high admission rate of sepsis (community-acquired or healthcare-associated) and as postoperative care prophylaxis, and all this leading to antibiotic resistance in critically ill patients [17]. However, adherence of medical practitioners with evidence-based guidelines of antibiotics had been strongly associated with improved patient progress and overall health outcome [18]. In addition, a number of studies have reported improved patient outcomes in terms of reduced risk of postoperative wound infection with the use of surgical antibiotic prophylaxis as per recommended guidelines [19–21].

Recently published studies have shared the high frequency of inappropriate antibiotics use [22] and ineffective ASPs in the hospitals of Pakistan with an inadequate multidisciplinary approach and lack of involvement of clinical leadership and trained individuals [23]. Furthermore, considerable challenges and resistance are faced in the actual clinical implementation of ASPs. Thus, the success of ASP may vary in different settings. There are fears among the frontline staff that ASP may lead to the delayed prescription of ‘‘potent’’ antibiotics, subsequently, high risk of developing septicemia, ICU admissions, prolong hospital stay, and mortality. Financial consideration, especially in the LMICs, is another important hurdle to implement ASP as it involves a significant human cost to run the program.

However, despite all the financial limitations, we intervened to implement ASP in an adult-SICU with administrative arrangements by the hospital administration to evaluate the feasibility of implementing ASP, the cost-effectiveness, and clinical impacts in a tertiary care hospital of Karachi, Pakistan.

## Methods

### Study design, settings, and duration

The impact of ASP implementation on predefined outcome measures was evaluated in the adult SICU of Aga khan university hospital (AKUH), a teaching tertiary care hospital in Karachi, Pakistan, and associated with Aga Khan University. The AKUH is catering to a large metropolitan city of a country with a population of 14.91 million.

We conducted a single-center quasi-experimental study and compared four-months (September to December 2017) of pre-ASP data with four-months (April–July 2018) of post-ASP data [24]. With three months (January to March 2018) as the implementation/training phase. During this phase, all the standard operating procedures (SOPs) for ASP were designed. The study protocol was approved by the institutional Ethical Review Committee (ERC) and waived the need for written informed consent for this retrospective study.

### Sample size and population

The sample size was calculated using PASS version 11 by considering the appropriateness of antibiotic use as a primary outcome. Literature suggested that after ASP driven intervention, the appropriate use of antibiotics for surgical prophylaxis improved from 78.1 to 88.4% [25]. We assumed a 15% increase in the appropriate use over the time with implementation of ASP in our SICU. To detect this difference sample size of 115 subjects required at each time point at alpha 5% and 80% power. All the patients admitted to adult SICU during the study period and prescribed any antibiotic for surgical prophylaxis or perceived clinical infection were included in the study.

### Date collection

Data was retrieved retrospectively, for patient demographics, comorbidities [26], antibiotics indication, surgical-specialty, and appropriateness of antibiotics prescriptions from the patient record of daily progress notes, observation charts, pathological, radiological, and microbiological reports. All the data was collected by infectious disease pharmacist (ID-Pharmacist), with the help of microbiologists, physicians, and nurses. All data collected on a structured data collection sheet. The online pharmacy system was used to verify the data of antibiotics therapy including the initiation date/time, route, dose, and total duration of therapy. Readmission and mortality data were retrieved from the SICU unit record datasheet.

To evaluate the economic impact, data was collected about the antibiotic’s prescription numbers, the number of unplanned readmissions, the cost of antibiotics, the direct and indirect cost of running ASP in the SICU. The per-hour cost of physicians, pharmacists, and nurses was noted from their monthly salary slip. In the case of readmission, none of the pre-ASP intervention phase patients was planned to include in the post-ASP intervention phase.

### Implementation of ASP

#### A multidisciplinary team and hospital administrative support

A multicomponent and multidisciplinary team driven ASP was implemented in 9 bedded open-multidisciplinary adult-SICU of AKUH in April 2018. ASP was introduced with the collaboration of pharmacy, surgery, critical care, infectious disease, and microbiology departments. Accordingly, the team members of this program were intensivist, infectious disease (ID)-trained pharmacist, and critical care nurse. Moreover, a microbiologist, an ID-physician, and a fellow were specially assigned from their respective departments after making SOP to take the agreement of all the stakeholders. Basically, ASP was led by an intensivist and ID-trained pharmacist. Administrative support was provided by the heads of all mentioned departments and hospital management in terms of workflow rearrangement and manpower redeployment to maintain the sustainability of the intervention. ASP implementation in SICU was approved by the hospital’s chief executive officer.

#### Prior authorization for restricted drugs

A restricted antimicrobials policy already implemented in the AKUH which requires approval from ID pharmacist/fellow. Restricted antibiotics include linezolid, tigecycline, fosfomycin, co-trimoxazole, and caspofungin, while colistin requires approval after 72 h. The common perioperative antimicrobials used in our SICU are cefazolin, ceftriaxone, and metronidazole. For postoperative complications, empirical antimicrobials are used which include, piperacillin-tazobactam, meropenem, imipenem, vancomycin, and colistin.

#### Prospective-audit-with-feedback

During the ASP team round in SICU, the team reviewed the antibiotics prescription appropriateness and source-control-documentation. Infectious disease physicians offered feedback on antibiotics prescriptions and microbiologists shared antimicrobial susceptibility patterns for appropriate antibiotic selection for different pathogens. Through prospective audit and feedback-mechanism changes were made and recorded on the structured data collection sheet by the pharmacist. Other ASP principles such as intravenous (IV) to oral (PO) conversion, de-escalation of empiric therapy based on culture results, and antimicrobial optimization were used to make recommendations to improve “appropriateness”. Antimicrobial optimization involved recommendations to improve the drug, dose, or duration of the antimicrobial based on patient characteristics, causative organism, site/type of infection, and pharmacokinetic/pharmacodynamics characteristics. Potential interventions of pharmacist and recommendations of the ASP team were then documented on patient progress notes.

#### Supplemental efforts

Along with the main intervention of ASP, some supplemental efforts were the part of this program, such as training of ID-specialist pharmacist, educational sessions for physicians, and source-control-documentation by the surgeons. Based on AKUH's developed antibiotics guidelines, different interventional strategies were used to implement ASP. These local guidelines describe the optimal choices of antibiotics along with doses and duration in treating infections. Training sessions were conducted to update SICU surgeons about the latest development in institutional antibiotic guidelines for adult surgical patients and they were also involved for the antibiotics follow up even when patients shifted from SICU.

Over the weekend and public holidays, the intensivist was responsible to take advice from the ASP team through telephonic communication. Infection control and prevention measures were strictly followed by the health care providers, such as contact isolation and hand hygiene.

### Outcome measures

The outcome measures of this study included specific antibiotic DDDs/1000 patient-days in the stewardship period, appropriateness of antibiotic prescriptions, mean duration of antibiotic, unplanned readmission, and mortality within 30-days of discharge from SICU and cost-effectiveness.

### Operational definitions

Defined daily dose (DDD) is the assumed average maintenance dose per day for a drug used for its main indication in adults. DDD is calculated by the following standard formula (27).

$${\text{DDD}}/{1}000{\text{ patient days}} = {\text{total consumption }}\left( {\text{gram or milligram}} \right){\text{ of drug}}/{\text{WHODDD patient days}}*{1}000$$

$${\text{Patient days}} = {\text{Number of days }}*{\text{occupancy }}\left( {\text{number of patients}} \right).$$

Mean duration is defined as the mean days that a patient received antibiotics during the study period. Source-control-documentation is defined as the post-surgery documentation of infection source control by the surgeons. Source control involves all actions undertaken to eradicate the infection source, reduce the bacterial inoculum, and restore normal physiologic function through correcting anatomic derangements [28]. Clinical indications for antibiotics in SICU are further divided into prophylactic antibiotics use for 24–48 h, empirical antibiotics therapy of 5–7 days for a post-operative complication, and targeted therapy of antibiotics in case of culture-proven infections [29].

Unplanned readmission in SICU was defined as readmission within 30 days after discharge. Patients were followed through the hospital admission record. In case of no readmission record found in our hospital, then on the 31st day of discharge patients were followed up by the study pharmacist on the provided contact number, to know about the readmission in any other hospital. Mortality within 30-days of discharge from SICU was monitored. For this purpose, patients were followed up by the study pharmacist on the provided contact number on the 31st day of patient discharge from the hospital. The record of readmission and mortality was maintained in the SICU ward record datasheet in both the phases. Appropriateness of antibiotic prescriptions was checked through monitoring (i) selection of right antibiotic/antibiotics to target pathogens (ii) use of most optimum doses (iii) blood levels concentration monitoring (iv) drug-interactions and (v) de-escalation /discontinuation (stop or change of the drug based on definitive diagnosis after 48-h [8].

### Data analysis

The mean (± SD) was calculated for continuous variables while categorical variables were presented as frequencies. All the recorded data related to basic demographic, relevant clinical variables, start and stop date of antibiotics, recommendations, compliance of ASP was analyzed using STATA version 15 (STATA Corp, Texas). To compare DDDs/1000 patient-days and mean duration of antibiotics and other parameters of pre-ASP and post-ASP the independent sample t-test and Mann–Whitney test were used for normally and not-normally distributed continuous variables, while *χ*^2^ was used for categorical variables. The variables were significant at *p* < 0.01.

## Results

A total of 123 patients enrolled in the pre-ASP period and 125 patients in the post-ASP period. Patients in both groups had an insignificant difference in the median age, gender, comorbid conditions, clinical indications for antibiotics, and surgical specialty involved (Table 1). None of the pre-ASP phase patients was there in the post-ASP phase.

**Table 1:** Patients’ characteristics and clinical indications for antibiotic in pre-ASP and post-ASP phases-ASP

Variable	Pre-ASP intervention (*n* = 123) (%)	Post-ASP intervention (*n* = 125) (%)	*p* value
Demographic characteristics			
Age (years)*	51 (11.2)	53 (9.5)	0.87
Gender (male)	88 (72)	93 (74)	0.68
Comorbidities**			
Cardiovascular illnesses	21 (17)	23 (18)	0.86
Chronic hepatobiliary illnesses	12 (10)	13 (10)	
Chronic renal illnesses	8 (7)	7 (6)	
Diabetes mellitus	30 (24)	32 (26)	
Malignancies	0	0	
Chronic neurological/neuropsychiatric illnesses	3 (2)	4 (3)	
Chronic respiratory illnesses	5 (4)	4 (3)	
≥ 2 chronic medical illnesses	8 (7)	7 (6)	
No comorbid condition	36 (29)	35 (28)	
Clinical indications for antibiotics in SICU			
Prophylaxis	58 (47)	55 (44)	0.78
Empirical	43 (35)	45 (36)	
Therapeutic	22 (18)	25 (20)	
Surgeries specialty involved			
General surgery	53 (43)	56 (45)	0.75
Neurosurgery	42 (34)	40 (32)	
Orthopedic	15 (12)	18 (14)	
Obs/gyn	7 (6)	6 (5)	
ENT	4 (3)	3 (2)	
Vascular	2 (2)	2 (2)	

*SICU* surgical intensive care unit, *ASP* antibiotic stewardship program,*Obs/gyn* obstetrics and gynecological, *ENT* ears, nose and throat, *ns* not significant

^*^Mean (SD)

**Comorbidities were coded from the World Health Organization, International Classification of Diseases, 10^th^ revision, Clinical Modification (ICD-10) [23]

Table 2 shows ceftriaxone, cefazolin and metronidazole were the most used prophylactic antibiotics. Carbapenem, piperacillin/tazobactam, vancomycin, and colistin were the most used empirical and therapeutic antibiotics. DDDs/1000 patient-days of all the antibiotics reduced in the post-ASP period, statistically significant reduction was found in ceftriaxone, cefazolin, metronidazole, piperacillin/tazobactam, and vancomycin. In the pre-ASP phase, ceftriaxone had DDD/1000 patient-days = 450 that reduced to 120 and for cefazolin 228/1000 patient-days reduced to 60/1000 patient-days. The duration of all antibiotics was reduced significantly (*p* < 0.01). In addition, the length of stay in SICU, the number of unplanned readmissions, and mortality reduced in the post-ASP period but statistically insignificant.

**Table 2:** Comparison of antibiotics use in pre-ASP and post -ASP phases

Variable	Pre-ASP intervention (*n* = 123)	Post-ASP intervention (*n* = 125)	*p* value
Total number of antibiotics doses	4400	2840	< 0.01
DDDs/1000 patient-days^*^			
Total antibiotics used	368	175	< 0.01
Prophylactic antibiotics			
Ceftriaxone	450	120	< 0.01
Metronidazole	280	110	< 0.01
Cefazolin	228	60	< 0.01
Empirical and therapeutic antibiotics			
Carbapenems	540	420	0.23
Piperacillin/tazobactam	390	150	< 0.01
Vancomycin	320	190	< 0.01
Colistin	310	240	0.24
Mean duration of antibiotics therapy (days)**			
Prophylactic antibiotics			
Ceftriaxone	5.5 (2.3)	1.8 (1.3)	< 0.01
Cefazolin	4.3 (2.7)	1.9 (1.4)	< 0.01
Metronidazole	3.0 (2.2)	1.5 (0.7)	< 0.01
Empirical and therapeutic antibiotics			
Piperacillin/tazobactam	6.7 (3.4)	2.7 (1.2)	< 0.01
Carbapenem	10 (3.6)	4.4 (2.3)	< 0.01
Vancomycin	6.5 (3.7)	3.1 (1.7)	< 0.01
Colistin	7.9 (4.6)	4.8 (2.6)	< 0.01
Other clinical measures			
Length of stay in SICU (days)**	5.2 (3.1)	4.7 (3.0)	0.09
Mortality	21 (17.1%)	18 (14.4%)	0.67
Readmissions	3.0 (2.4%)	1.0 (0.8%)	0.07

^*^Defined daily dose/1000 patient days; **data presented as mean (SD); SICU = surgical intensive care unit; ASP = antibiotic stewardship program

Table 3 demonstrates that the duration of therapy (> 5 days) was found in 76% of patients in the pre-ASP phase while only 14% of patients in the post-ASP phase. The selection of antibiotics as per guidelines also improved significantly (*p* < 0.01) in the post-ASP phase. While Source-control-documentation by surgeons and ID trained pharmacist involvement was not present in the pre-ASP phase. In the post-ASP phase, pharmacist interventions were made in 62% cases and source-control-documentation by surgeons was completed in 50% of patients. Table 4 summarized the significant improvement in all the parameters of the appropriateness of antibiotic use in the post-ASP phase.

**Table 3:** Comparison of interventions data in pre-ASP and post-ASP phases

Variable	Pre-ASP intervention (*n* = 123) (%)	Post-ASP intervention (*n* = 125) (%)	*p* value
Pharmacist interventions	None	78 (62.4)	
Selection of antibiotic as per guidelines	52 (42)	94 (75)	< 0.01
Duration of therapy > 5 days	93 (76)	17 (14)	< 0.01
Source-control-documentation	None	62 (49.6)	–

*ASP* antibiotic stewardship program; *Pharmacist interventions* dose optimization/drug interactions/levels monitoring

**Table 4:** Appropriateness of antibiotic prescriptions in pre-ASP and post-ASP phases

Parameters of appropriateness of antibiotic use	Pre-ASP intervention (*n* = 123) (%)	Post-ASP intervention (*n* = 125) (%)	*p* value
Selection of right antibiotics to target pathogens	52 (42)	94 (75)	< 0.01
Use of most optimum doses	30 (24)	109 (87)	< 0.01
Blood levels concentration monitoring	15 (12)	100 (80)	< 0.01
Drug-interactions	32 (26)	15 (12)	< 0.01
De-escalation/discontinuation of antibiotics based on definitive diagnosis after 48-h	25 (20)	120 (96)	< 0.01

Single patient might appear in no, one or more than one parameter

Table 5 shows the detail of ASP related cost breakdown in both the phases. Table 6 summarized the ASP related cost. The net cost saving is 6360 US$ per year in 9 bedded SICU. The major cost-saving was noted due to reduced antibiotics consumption, around US$ 18,000 (PKR 2.8 million) yearly.

**Table 5:** Antibiotics stewardship programme (ASP) related cost breakdown in pre-ASP and post-ASP phases

Variable	Pre-ASP intervention			Post-ASP intervention		
	Unit cost (US$)	Quantity	Total cost (US$)	Unit cost (US$)	Quantity	Total cost (US$)
Direct cost						
Physician cost (hours)	–	–	–			
Education Consultation Feedback				25	180	4500
Microbiologist cost (hours)	–	–	–			
Data collection Susceptibility reporting				12	90	1080
Pharmacist cost (hours)	–	–	–			
Data collection and analysis Interventions				5	215	1075
Administrative cost for ASP designing, implementing, monitoring and evaluating (hours)	–	–	–	30	50	1500
Indirect cost						
Pharmacy time (hours)						
Pharmacist order processing	3	200	600	3	80	240
Technician (preparation and dispensing	2	400	800	2	190	380
Medication administration nurses time (hours)	2.5	750	1875	2.5	396	990
Consumables and logistics related to IV ABXs administration	1.5	4400	6600	1.5	2840	4260
SICU bed per day cost	540	5.2*	2808	540	4.7*	2538

^*^Mean length of SICU stay (day); The same brands of all the antibiotics were purchased and used throughout the study in pre and post phases

**Table 6:** Summary of ASP related cost in 4 months period in SICU unit (*n* = 125)

Variables	Post-ASP Implementation	Pre-ASP Implementation	Net cost saving
Direct ASP costs (US$)	8155	0.0	8155
Indirect ASP costs (US$)	8408	12,683	− 4275
ABXs cost/4 months (US$)	14,000	20,000	− 6000
Net ASP cost/4 months (US$)	30,563	32,683	− 2120
Net ASP cost/year (US$)	91,689	98,049	− 6360

The same brands of all the antibiotics were purchased and used throughout the study in pre and post phases

## Discussion

The strength of this ASP was the execution of main interventions of ASP i.e. prior authorization for restricted drugs and prospective-audit-with-feedback along with the supplemental efforts of training pharmacists, updating physicians, and source-control-documentation by the surgeons. We found this dual effort very effective like previous studies [30]. Moreover, the results of our study showed a robust and significant impact of ASP on antibiotic use in our adult SICU. Considerably in the pre-ASP phase, there was poor adherence to antibiotics guidelines in terms of duration of therapy and appropriate antimicrobials selection. In the post-ASP phase duration of targeted therapy of fewer than 5 days and the selection of appropriate antibiotics improved significantly. Almost 50% reductions in the use of prophylaxis and empirical antibiotics were achieved. This remarkable ASP implementation impact could be related to the ID-trained pharmacist involvement in the ASP-team that contributed through making valuable intervention of drug interaction, therapeutic serum concentration monitoring, and doses optimization in 62% cases. Prospective-audit-with-feedback decreased delays in antibiotics prescription, dispensation, and administration.

There were 3 unplanned readmissions in the pre-ASP phase and all related to infection while in the post ASP phase there was only one unplanned readmission, but not due to infection. Shorter SICU stay was observed in the post ASP phase, which could better indicate the impact of ASP with multiple strategies to reduce the infection-related SICU stay. Our results are also supported by previous literature [9, 21, 31].

Several published ASP reports have shown incontestable vital and firm effects on antimicrobial usage with reduced healthcare-associated infection rates and mortality [15]. However, targeted clinical indications of antibiotics in our study were almost the same in both phases. A recent meta-analysis reported the irrational use of antimicrobials in intensive care patients [32], which is a key factor of the increasing trend of ABX-R. Similar practices of longer duration of antibiotics use were found in our SICU setting in the pre-ASP phase. However, in the post-ASP phase statistically significant reduction in the mean duration of antibiotics was recorded. Nevertheless, our study is conducted in the region of increasing ABX-R, we found higher mortality rates (21%) in patients receiving improper antimicrobials in pre-ASP. Similar results are reported by previous studies, with a high mortality rate at 28 and 60 days in patients receiving inappropriate antibiotics [32]. Therefore, in the four-month post-ASP period, we found a reduced mortality rate (13%) within 30 days follow up from a day of discharge.

The lack of adherence to ASP principles by the surgical teams also affects the medical care of the patients. Historically ASP efforts have mainly targeted medical specialties [33]. This is a critical oversight, and optimizing antimicrobial prescribing before, during, and after surgery should be a central principle of tackling antimicrobial resistance [34]. Most of the ASP research in surgery is focused on antibiotic prophylaxis and prevention of surgical site infections [33]. It is imperative however to engage more with surgical teams and try and address antimicrobial prescribing more comprehensively along the entire surgical pathway. It has previously been reported [35–37] that peer endorsement is an important factor in the uptake of sustainable interventions in healthcare. The role of local champions and organizational leaders is important determinants in shifting antimicrobial prescribing behaviors, social norms, and values over time.

To address and overcome this lapse, source-control-documentation was used as one of the main components of the ASP, as documentation of the rationale of antimicrobials at the start is associated with better outcomes in terms of de-escalation or discontinuation [38]. In the post-ASP phase, source-control-documentation improved up to 49.6%, with the help of educational sessions conducted in the implementation phase before starting ASP in SICU. Therefore, improvement in source-control-documentation helped in the timely de-escalation or discontinuation of empirical antibiotics, which have a significant effect on alleviating the emergence of resistance [39].

Some healthcare providers consider that an ASP emphasizes more on cost savings rather than patient care and quality improvement. This study in SICU of LMIC showed that with ASP implementation all the clinical measures improved. Major cost saving resulted due to a reduction in inappropriate consumption and expenditure of antibiotics via optimal selection and prescription, the ultimate target of ASP implementation [9]. All the extra human costs of our ASP were also compensated by savings from antibiotics expenditure. We found that the physician’s consultation and feedback cost constituted the biggest part of all ASP direct costs. Human cost reduction and further improving the savings from antibiotics expenditure is required for the sustainable success of ASP. It needs to cultivate correct prescribing behavior among the doctors through continuous education and timely feedback mechanism. The administrative arrangements have been made formally with the involvement of the hospital administration; thus the continuity of the practices will be audited that will give a sustained impact.

Overall with the implementation of ASP in our SICU, a hospital can possibly save around US$ 18,000 (PKR 2.8 million) yearly from the rational use of antibiotics, which is a significant figure. To our knowledge, it is the first report from SICU of any LMIC and has shown remarkable achievement in terms of antibiotics consumption, clinical and economic outcomes.

The findings of the current study from tertiary care private sector teaching hospital in Karachi, Pakistan encourages the public sector hospitals to identify key champions to engage with government and policymakers to ensure effective support for and scale-up of interventions in the public sector hospitals to implement the ASP program [40]. To optimize antimicrobial use and establish effective ASP in LMICs settings first there needs to be equitable access to standardized antimicrobials across the entire healthcare pathway [41]. This raises the question of the ethics of restricting excess antimicrobial use through ASP, in settings where inadequate access to healthcare remains a key issue. There must be a balance between reducing excess antimicrobial use without impeding access to them. ASP is essentially about optimizing antibiotics use, and it is therefore critical that it should not impede access to vulnerable populations in LMICs [42].

In developed countries, the healthcare expenditure and investments by the government are accompanied by externally driven targets and performance measures which can put a strain on diminishing resources [42]. The use of an interdisciplinary approach, especially the involvement of ID trained specialized pharmacists is the key determinant of success in implementing the ASP program in our setting that can overcome some of the resource limitations. The success of this primarily ID pharmacist-led ASP initiative provides learning for other resource-limited settings [43]. The direct involvement of the surgical team through the source control documentation is another important determinant of success that addressed antimicrobial prescribing more comprehensively along the entire surgical pathway [35–37].

The limitations of our study include; it is a single centered study in private sector tertiary care hospital with limited duration and generalizability. The quasi-experimental design is associated with inherent limitations, including the potential for confounding bias. However, we did not find a significant difference in the patients’ characteristics and clinical indications for antibiotics in pre-ASP and post-ASP phases. Still, differences in unmeasured factors may exist between the groups. Although ICU is not in the first line of health care systems that suffer a remarkable seasonal effect for the antibiotic prescription, there was a seasonal difference in the pre and post-analysis period in this study. Non-compliance by the surgeons with source-control-documentation was observed, that might overcome with educational sessions. ASP-team could not follow the patient shifted from the SICU due to limited human resources. Nonetheless, our study could serve as an example to other LMICs stewardship programs that are interested in analyzing the potential effectiveness of their interventions.

### Conclusions

We can summarize that implementation of ASP with supplemental efforts can improve the appropriateness of antibiotics prescriptions and optimization of the duration of antibiotics use. This approach is cost-effective mainly due to the reduced cost of antibiotics with rational use. Source-control-documentation is very important in surgical patients, its 100% compliance can improve the antibiotics practices to minimize the antimicrobial resistance in surgical patients. We also conclude that ASP implementation is feasible as only a comparatively few key healthcare providers are involved. The generalisability and sustainability of ASP to other medical units, acute care units, and outpatient clinics could be investigated in future studies.

## Data Availability

All data generated or analyzed during this study are included in this published article. The datasets used and/or analyzed during the current study are available from the corresponding author on reasonable request.

## Abbreviations

ABX-R: Antibiotic resistance
LMICs: Lower/middle-income countries
ICUs: Intensive Care Units
SICU: Surgical Intensive Care Unit
PICU: Pediatric Intensive Care Unit
ASP: Antibiotic Stewardship Program
DDD: Defined Daily Dose
SHEA: Society for Healthcare Epidemiology of America
IDSA: Infectious Diseases Society of America
ID-Pharmacist: Infectious Disease Pharmacist
WHO: World Health Organization
